# Evidence for murine cutaneous immune surveillance localized to hair follicle epithelium

**DOI:** 10.3389/fcell.2026.1721181

**Published:** 2026-04-24

**Authors:** Diana Del Castillo, Hannah Kim, Sumaya Troy Alaama, David D. Lo

**Affiliations:** Division of Biomedical Sciences, University of California Riverside School of Medicine, Riverside, CA, United States

**Keywords:** antimicrobial peptide, epidermis, gram-positivebacteria, hair follicle, innate immunity

## Abstract

Epithelial expression of the gene encoding the anti-microbial peptide PGRP-S was previously found to be specifically associated with M cells in mucosal epithelium. However, recent studies have found PGRP-S expression by a variety of epithelia not providing direct M cell-like microparticle transcytosis into lymphoid tissues. Thus, in mouse epidermis we found that PGRP-S expression, revealed by a dsRed reporter, was specific to subsets of hair follicle epithelium, depending on the specific skin region, and not associated with lymphoid aggregates. Interestingly, expression in flank skin hair follicle epithelium was also transiently inducible upon exposure to a suspension of *Staphylococcus aureus*. Altogether, the topology of hair follicles and location of dsRed-expressing epithelia are consistent with a model in which this PGRP-S-dsRed reporter identifies a population in hair follicles associated with surveillance of environmental exposures.

## 1 Introduction

Across all barrier tissues, the epithelium serves as the foremost boundary between host and environment. These tissues face constant exposure to microbes, antigens, toxins, and physical insult yet they must coordinate necessary barrier and physiological functions such as nutrient absorption, gas exchange, and sensory perception with appropriate immune responses. Importantly, epithelial beds are not uniform; barrier tissues house specialized niches that can affect immunological functions, especially with regard to immune surveillance. The gut is commonly simplified to consisting of enterocytes that are responsible for intake of nutrients, however, the hills and valleys of villi and lymphoid follicles yield locally discrete and diverse populations of epithelia (Haber et al., 2017). Similarly, the lungs differ in epithelial populations at different levels along the respiratory tracts, changing in composition from proximal to distal airways and into the alveolus (Meng et al., 2023). This diversity and specialization of epithelia allow barrier structures to meet the structural, metabolic, sensory, and crucially, the immunological demands of the local microenvironment.

The skin is the largest epithelial barrier, forming a critical interface between the internal and external environment. Beyond its structural role, the skin functions as a dynamic immunological organ that integrates physical defenses, biochemical secretions, and resident microbial communities to uphold homeostasis (Chen et al., 2018; Zhang et al., 2022). This complex ecosystem is under constant challenge by microbial exposure and mechanical injury, requiring precise regulation of innate defense mechanisms to maintain barrier integrity. Hair follicles (HFs) represent discrete anatomic niches that shelter colonizing organisms, as well as provide a focus for localized host responses (Paus et al., 1998; 2003; Kiselev and Park, 2024). Therefore, we infer that HF epithelium responds differently than interfollicular epithelium in the context of microbial challenge; the question, however, is how to precisely assess such induction.

Peptidoglycan recognition proteins (PGRPs or PGLYRPs) have been historically appreciated for their function and conserved nature in mammalian innate immunity. This family of proteins consisting of PGRP-S, PGRP-L, PGRP-Iα and PGRP-Iβ (also referred to as PGLYRP1-4 respectively), have been shown to mitigate pathogen-interactions through sensor and effector functions. Their ability to detect peptidoglycan, a key component of the bacterial cell wall, and trigger (or participate in) downstream antimicrobial responses makes them key frontline players (Dziarski and Gupta, 2010). Moreover, they appear within unique anatomical niches. While PGRP-L, has been mainly associated with its appearance in the liver and PGRP-Iα and PGRP-Iβ are expressed in barrier tissues such as the skin, mucosa and secretions, PGRP-S has been notably associated with its expression in neutrophils (Dziarski and Gupta, 2006; 2010; Lu et al., 2006). This immune cell-antimicrobial protein relationship has been well characterized (Liu et al., 2000; Cho et al., 2005). However, the expression and function of PGRP-S in epithelial compartments is less understood.

Using a transgenic reporter that encodes for the fluorescent protein dsRed under the control of the PGRP-S promoter (PGRP-S-dsRed mouse), we established a reporter to reveal regulation of this gene *in vivo* and used this to investigate expression in various epithelial populations (Wang et al., 2011; Del Castillo and Lo, 2024). Notably, PGRP-S was discovered to be expressed in the specialized epithelial cells known as M cells, with specific expression in intestinal Peyer’s patch follicle epithelium, nasopharyngeal associated lymphoid tissue epithelium (NALT), lung bronchus-associated lymphoid tissue epithelium (BALT), and even in a subset of thymic medullary epithelium (Wang et al., 2011). Interestingly, in these specialized epithelial cells, PGRP-S-dsRed expression was not associated with antimicrobial function; these epithelial barrier M cells frequently serve as conduits for entry of pathogenic microbes across the mucosal barrier into the body without any antimicrobial action within the M cells themselves (Jensen et al., 1998). These findings underscore the presence of a common phenotypic program with unique specialized mechanisms of epithelial monitoring and defense in the service of maintaining a selective barrier function. In the present study, we extended our analysis to the epidermis and discovered additional instances of constitutive expression of PGRP-S-dsRed in specific epithelial subpopulations as well as in response to immune stimuli.

## 2 Materials and methods

### 2.1 Mice and transgene construction

Mixed sex cohorts of C57BL/6J mice ages 8–15 weeks were obtained from The Jackson Laboratory (Sacramento, CA) All mice were housed and bred in a specific pathogen-free vivarium at the University of California, Riverside, prior to experimental use. PGRP-S-dsRed transgenic mice, previously generated in our laboratory by targeting the *PGLYRP-1* locus, were bred on the C57BL/6J background and used in the present study (Wang et al., 2011). CX3CR1-eGFP reporter mice were also utilized where the endogenous CX3CR1 promoter drives eGFP expression, therefore only heterozygous mice were used. Double transgenic mice were created by breeding individuals positive for each transgene. TNFdARE were graciously provided by Dr Fabio Cominelli at the Case Western Reserve University. TNFdARE/PGRP-S-dsRed + mice were generated by backcrossing with PGRP-S-dsRed mice and sacrificed at ages 5–7 weeks and used as TNFdARE heterozygotes. All animal studies were conducted in compliance with the campus Institutional Animal Care and Use Committee (IACUC) (Animal Use Protocol #40), ARRIVE guidelines, and National Institutes of Health (NIH) guidelines.

### 2.2 Bacterial, cultures and topical treatment

*Staphylococcus aureus* strain [Strain ID: LAC-13C; Plasmid: pCM29], generously provided by Dr. Tammy Kielian from the University of Nebraska Medical Center, expresses green fluorescent protein (GFP). Bacterial cultures were grown in LB broth supplemented with 10 μg/mL of chloramphenicol at 37 °C for 12–16 h until reaching an optical density at 600 nm (OD_600_) of approximately one for use in topical treatments. *Staphylococcus aureus* was handled in accordance with an approved protocol in a BSL-2 laboratory space. Mice were anesthetized via intraperitoneal injection of ketamine (100–120 mg/kg) and xylazine (14–18 mg/kg). Following anesthesia, a 1 × 2 cm area of dorsal skin was shaved. Treatments consisted of topical application of 100ul of bacterial suspension to the shaved area All mice were singly housed during experiments.

### 2.3 Skin tissue collection and processing

Mice were euthanized via isoflurane anesthesia followed by cervical dislocation. Dorsal skin and whiskers was excised and fixed in 4% paraformaldehyde at 4 °C for 12–16 h. Samples were cryoprotected in 30% sucrose (in PBS) at 4 °C for at least 24 h until fully saturated. Skin was embedded in OCT compound and cryosectioned at a thickness of 25 µm. For circumvallate papillae and lung, samples were only fixed for 30 min before cryoprotection in sucrose and snap frozen in OCT in liquid nitrogen, respectively.

### 2.4 Imaging and quantification

Sections were collected from samples frozen in OCT, counterstained with DAPI, and mounted in Prolong Gold Antifade medium (ThermoFisher, cat#P36930). For immunofluorescence, lung sections were fixed for 20 min in 4%PFA and stained with anti-B220 antibody conjugated to AlexaFluor488 (Biolegend, cat#103225) and anti-CD4 antibody conjugated to AlexaFluor647 (Invitrogen, cat# MCD0421) at 1:1000 and 1:500 dilutions, respectively. Whisker and dorsal skin sections were subjected to antigen retrieval with Tris EDTA, permeabilized with TritonX-100 and blocked with 10% normal goat serum. The primary antibody, rabbit anti-mouse SpiB (Bioss, cat# BS-17663), was utilized at a 1:200 dilution and incubated on slides overnight at 4 °C. Slides were washed and incubated with a AlexaFluor647-conjugated goat anti-rabbit secondary (Invitrogen, cat# A21245) for 1h at room temperature Slides were counterstained and mounted as described above. Images were acquired using a Yokogawa spinning disk confocal microscope using identical acquisition settings for all samples. For each mouse, 10 representative fields were collected from 2-3 tissue sections, yielding 100–150 images per mouse. Each experimental group consisted of at least three biological replicates, resulting in a total of 300–450 images per condition.

Induction of ds-Red in hair follicles was quantified by counting the number of all PGRP-S+ follicles and sebaceous PGRP-S+ positive follicles per image. Statistical analyses were performed in GraphPad Prism (version 10.6.1).

### 2.5 Neutrophil counts

Dorsal skin samples were collected from 3 mice per treatment group at 24 h, 72 h, and 1 week following *S. aureus* exposure, as well as from LB-treated control mice at 72 h. For each sample, 50 images were randomly acquired. Neutrophils were identified by PGRP-S-dsRed expression and morphology, as validated by Wang et al. (2011) and infiltration was quantified through direct counts per image. One-way ANOVA with *post hoc* Tukey’s and Kruskal–Wallis with *post hoc* Dunn’s tests were applied to PGRP-S percentage data and neutrophil counts respectively using GraphPad Prism (version 10.6.1).

### 2.6 RNA extraction and nanostring RNA profiling

RNA was purified from fresh frozen dorsal skin using a phenol–chloroform extraction method with TRIzol© (Ambion, Carlsbad, United States) based method. 50 ng of purified RNA was analyzed using an nCounter® Sprint Profiler (NanoString Technologies, Seattle, United States) with the nCounter® Mouse Myeloid Innate Immunity Panel according to manufacturer protocols. The nSolver® 4.0 software (NanoString Technologies, Seattle, United States) was used to normalize gene counts based on housekeeping genes and positive controls. Differential expression was calculated using the nSolver® Advanced Analysis 2.0 software (NanoString Technologies, Seattle, United States), and p-values were adjusted using the Benjamini–Hochberg method.

## 3 Results

### 3.1 PGRP-S reporter expression appears in M cells and similar specialized cells in other epithelia

We previously reported studies using a PGRP-S-dsRed reporter mouse on specialized cells including M cells in mucosal tissues as well as granulocytes, especially neutrophils (Wang et al., 2011; Del Castillo and Lo, 2024). However, characterization of this reporter in epidermal barrier tissue has not been explored. The generation of the M cell phenotype has mainly been in mucosal epithelium, derived from embryonic endoderm. Their induction is dependent on local cell interactions as well as specific cytokines. Consequently, they are found associated with organized lymphoid tissues such as Peyer’s patches in the small intestine and colon (Mabbott et al., 2013; Dillon and Lo, 2019). However, the M cell phenotype is not limited to intestinal epithelium. For example, in the thymus, a subset of M cell-like cells is found in the medulla (Givony et al., 2023; Del Castillo and Lo, 2024) also from endodermal origins.

**FIGURE 1:**
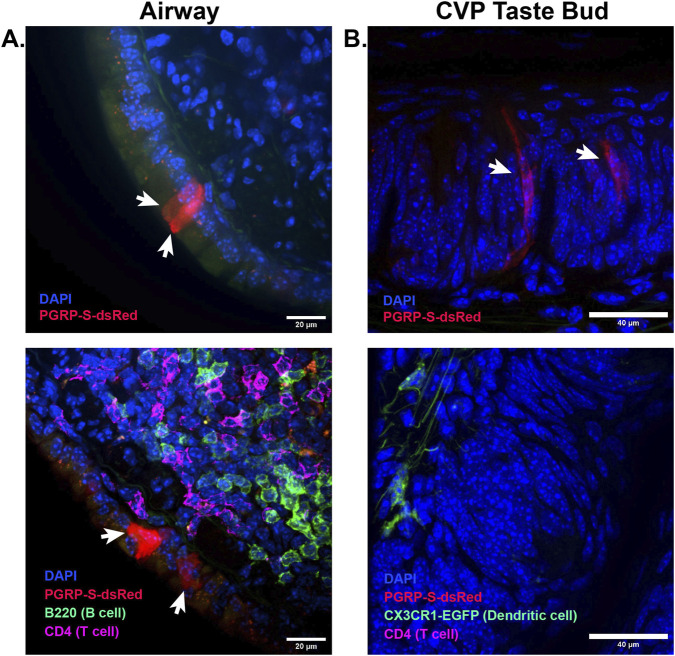
PGRP-S-dsRed expression in the lung and taste cells of the circumvallate papillae. **(A)** Representative images of PGRP-S-dsRed expression (arrows) in the airways of 4–6-week-old TNFa-overexpressing dARE mice (top) and immunofluorescence staining of PGRP-S-dsRed tissues to B cells and CD4 T cells showing association with bronchus-associated lymphoid tissue (BALT) (bottom). **(B)** Representative images of PGRP-S-dsRed (top, arrows) and CX3CR1-eGFP (bottom) expression using reporter mice in the taste bud of circumvallate papillae (CVP).

In tissues outside of the intestine, endoderm-derived bronchial epithelium can also give rise to PGRP-S-dsRed-expressing M cells associated with bronchus-associated lymphoid tissue (BALT (Kimura et al., 2019)) which we visualized in a TNF-overexpressing mouse model, TNFdARE (Figure 1A) (Kontoyiannis et al., 1999; Kimura et al., 2019). A similar cell type can also be found in the floor of the ectoderm-derived nasal cavity, associated with Nasopharyngeal Associated Lymphoid Tissue (NALT (Wang et al., 2011)). Thus, despite distinct embryologic origins, a familiar M cell phenotype can emerge to perform similar immune surveillance function(s). Interestingly, an unusual PGRP-S-dsRed-expressing cell is also found with a strikingly different morphology (within) the taste buds of the circumvallate papillae (Figure 1B). These cells appear to be the same as the taste bud cells shown to express SpiB and exhibit microparticle uptake function (Qin et al., 2023). Taste buds can be derived from either endoderm or ectoderm, indicating development of a convergent morphology and function. The morphology of the taste bud cells sets them apart from M cells described previously; they are not found near lymphoid aggregates or dendritic cells (Figure 1B) but their ability to capture microparticles may be helpful in accumulation of material from food, either to assist in taste (Qin et al., 2023)or for some unidentified immune surveillance function.

**FIGURE 2:**
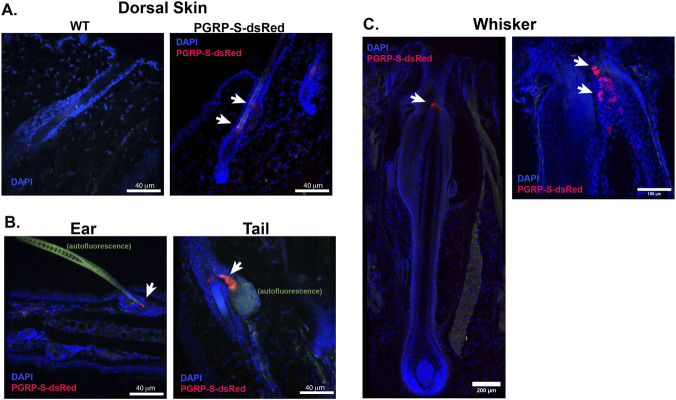
Cutaneous PGRP-S-dsRed signal is localized specifically to hair follicles but differs with anatomical location. **(A)** Naive WT and PGRP-S-dsRed dorsal skin hair follicles showing PGRP-S+ expression signature at baseline. **(B)** Representative images of other skin tissues showing the ear (left) and tail (right); arrows highlight the PGRP-S+ signal. **(C)** Representative images of whisker PGRP-S-dsRed expression in the whisker showing localization to the upper follicle (left) and a zoomed in image showing dsRed + cells distributed within the isthmus of the follicle. n = 3-6 mice.

With this context, we examined epidermis to determine whether a similar dsRed-expressing epithelial cell might be induced at sites where surveillance at the epithelial barrier may be required. Interestingly, PGRP-S reporter expression was found in several skin regions, always localized specifically to hair follicles and notably not in other, non-follicular regions of the epidermis (Figures 2A–C). To validate the specific dsRed signal, non-transgenic wild type (WT) animals from the same colony had no detectable dsRed signal, confirming that the fluorescence was not due to autofluorescence or artifact signals (Figure 2A). In each type of hair follicle, the location of the PGRP-S-expressing cells was slightly different (Figures 2A–C). In most skin regions, these cells were near or overlapping the sebaceous gland cells; since the PGRP-S peptide itself has antimicrobial activity, it may be part of the larger array of antimicrobial peptides constitutively secreted by sebaceous gland cells (Cho et al., 2005; Dziarski and Gupta, 2010; Zouboulis et al., 2022). None of the PGRP-S-dsRed expressing cells were directly associated with lymphoid aggregates nor dendritic cells.

### 3.2 Mouse vibrissae contain a constitutive population of PGRP-S-dsRed cells that share M cell-like characteristics

In the whiskers/vibrissae, the PGRP-S-dsRed-expressing cells showed a strikingly distinctive mesh pattern distribution surrounding the isthmus region of the hair follicle (Figure 2C). Vibrissae are known to be highly innervated, providing mechanosensory information to the somatosensory cortex (Takatoh et al., 2018),but it is unclear whether dsRed-expressing cells could provide any immune surveillance input to the somatosensory system. However, similarities to mucosal M cells may point toward a defined immune sensing role.

**FIGURE 3:**
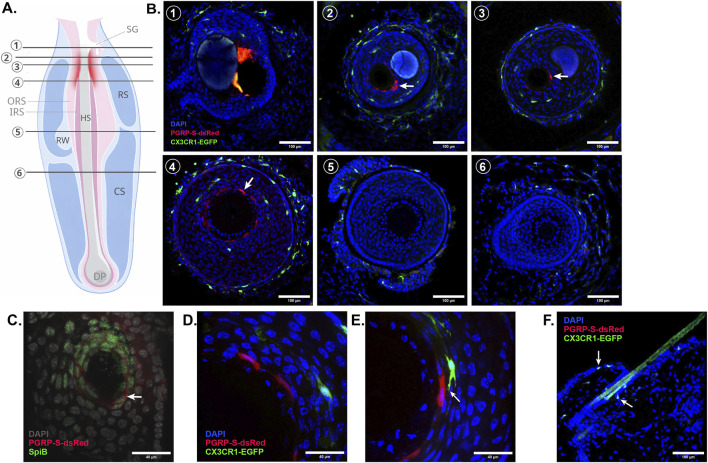
Whisker epithelia shows constitutive PGRP-S-dsRed + cell expression in isthmus with M cell-like features. **(A)** Model of murine vibrissae showing general location of PGRP-S-dsRed expressing cells in red as well as landmarks including the sebaceous gland (SG), hair shaft (HS), outer root sheath (ORS), inner root sheath (IRS), ring sinus (RS), ringwulst (RW), and dermal papilla (DP). **(B)** Transverse cross sections of whisker follicles (1–6) at depths shown in Figure 3A. **(C)** Immunofluorescence staining of PGRP-S-dsRed + whiskers with SpiB antibody showing colocalization of nuclear SpiB with cytoplasmic dsRed signals (arrow). **(D)** Zoomed in image of Figures 3B–4 showing concurrent PGRP-S-dsRed and CX3CR1-eGFP expression in the whisker follicle of naïve mice. **(E)** instance of direct contact between dendritic cell process with PGRP-S-dsRed + cell (arrow). **(F)** Representative images of CX3CR1-eGFP cell (arrow) distribution near hair follicle in dorsal skin. n = 2–6; 121 whisker follicle cross sections analyzed.

The anatomical location of these cells places them near the opening of the active follicle and interestingly, below the sebaceous gland in the isthmus region (Figures 2C, 3A,B). To more concretely identify this cell population, we performed immunostaining of SpiB, a transcription factor which regulates the M cell program (Kanaya et al., 2012), and found colocalization with dsRed + cells in the isthmus. The localization of both PGRP-S-dsRed + cells and CX3CR1+ cells is also seemingly grouped as they are noted to appear at similar locations along the length of the follicle (Figures 3A,B), with close proximity and possible direct contact—a relationship reminiscent of that seen in the mucosa (Figures 3D,E) (Sakhon et al., 2015). This contrasts with the CX3CR1 expression of the naïve flank skin (Figure 3F) which is far more sparse and possibly due to dendritic epidermal T cells in the epidermis or dermal antigen presenting cells (APCs) (Almeida et al., 2015; Tanaka et al., 2023).

### 3.3 Dorsal skin expression of PGRP-S-dsRed in HFs is inducible and shows distinctive kinetics

**FIGURE 4:**
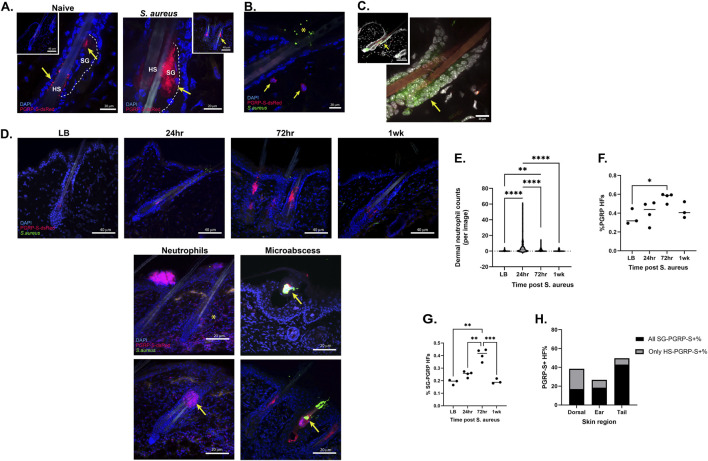
Topical S. Aureus to dorsal skin induces SG-associated PGRP-S expression with differing kinetics to neutrophil recruitment. **(A)** Zoomed in visualization of the different PGRP-S signals illustrate hair shaft (HS)-associated signal (arrow) with weak expression in sebaceous gland (SG) of naive skin (left), compared to sebaceous gland (SG)-associated induction (arrow) upon stimulation with *Staphylococcus aureus* (right). The dashed line delineates the sebaceous gland. **(B)** Representative confocal image of PGRP-S expression in skin neutrophils post *Staphylococcus aureus* application. Arrows point to two distinct neutrophils, and an asterisk indicates an aggregation of applied fluorescent *Staphylococcus aureus.* **(C)** Immunofluorescence staining of dorsal skin HF with SpiB. **(D)** Representative confocal images of post topical *Staphylococcus aureus* exposure at 24h, 72h and 1week with LB vehicle control for comparison to visualize hair follicle PGRP-S-dsRed (row 1), dermal neutrophil recruitment (row 2) and microabscess appearance (row 3). **(E)** Dermal neutrophil counts for the different timepoints, n = 3-4 mice per group; Kruskal–Wallis with *post hoc* Dunn’s for multiple comparisons. **(F)** HF count percentages showing PGRP-S-dsRed + signal; n = 3–4. One-way Anova with *post hoc* Tukey’s multiple comparison. **(G)** HF count percentages showing sebaceous gland-associated PGRP-S-dsRed + signal; n = 3–4. One-way Anova with *post hoc* Tukey’s multiple comparison. **(H)** Bar graph representation of pooled PGRP+ and SG-PGRP-S + HF (black) counts of different skin tissues, the remaining PGRP-S+ non SG-PGRP-S HFs are included in the grey bar labeled “only HS-PGRP-S”, n = 3-4 mice per group. For PGRP-S-dsRed+, Chi-square statistic, df: 31.07, 2; p < 0.0001. For SG-PGRP-S-dsRed+, Chi-square statistic, df: 63.34, 2; p < 0.0001.

While PGRP-S reporter expression was found in hair follicles in the ear, tail, whisker, and dorsal skin, closer inspection of the positive hair follicle-associated signal in naive mice revealed two distinct PGRP-S-dsRed fluorescence patterns. One expression pattern was a hair shaft-associated signal (HS-PGRP) which was bright, punctate, not contained within cells and located at the isthmus of the HF. This appears to be attributable to secreted dsRed protein adhered to the hair shafts. A second pattern was a sebaceous gland-associated signal (SG-PGRP) which was dimmer, broader, sometimes cell associated and located higher on the hair follicle structure at the SG junction (Figure 4A).

The striking distribution of PGRP-S-dsRed-expressing cells in hair follicles suggested a potential functional phenotype; keeping with the M cell motif, we considered whether these cells might have an immune surveillance function. Since the skin is usually colonized by and also actively infected by Gram-positive bacteria, we tested whether expression might be regulated by the addition of topical *S. aureus* (Lange-Asschenfeldt et al., 2011). We applied *S. aureus* to the shaved dorsal skin of PGRP-S-dsRed reporter mice and assessed expression. Following topical application of *S. aureus*, recruited neutrophils could be seen in the dermis of dorsal skin (Figures 4B,D) and served as a comparison point for the distinct expression in epithelia. As with PGRP-S-expressing follicle epithelium in whiskers, we found SpiB immunofluorescence staining in the dorsal skin specifically expressed in a subset of epidermal cells in the hair follicle. Importantly, as with the PGRP-S-dsRed signal, SpiB staining was specific to hair follicle epithelium (Figure 4C).

The sebaceous gland-associated expression of the PGRP-S reporter becomes significantly more evident with the application of *S. aureus* while HS-PGRPs remained unchanged (not shown). Under this condition, SG-PGRP (that is, the reporter dsRed fluorescence signal) is clearly associated with the HF-SG junction as seen by PGRP + cells and is established as a secretion showing expression continuing into the lumen of the HF (Figure 4A).

Expression of PGRP-S-dsRed was exclusive to HFs; therefore, we sought to identify the kinetics of this response by analyzing expression after 24 h, 72 h, and 1 week. The innate acute response was evident by neutrophil recruitment to the dermis of the skin which appeared to peak at the 24h timepoint. Analysis of tissue sections showed that 27% of images showed some level of neutrophil recruitment as compared to 0%–3% in all other groups, indicating complete resolution within 1 week. Direct neutrophil counts in tissue sections confirmed that infiltration peaked around 24 h (Figure 4E). After 72 h, neutrophils can still be found, however less so in the dermis and more frequently in microabscess type structures. Similar microabscess structures can be seen at 24 h s post exposure, though isolated *S. aureus* cells are often seen scattered across the epidermis. By 72 h most residual *S. aureus* appears to be encapsulated by secretions suggesting that they are aiding in clearing bacteria from the HF (Figure 4D).

Interestingly, the PGRP-S-dsRed response in hair follicles did not follow the kinetics of dermal neutrophil recruitment which appeared to peak at 24h; instead, the hair follicle SG response seemed to be highest at the 72h timepoint. Using LB vehicle controls at the 72h timepoint, we quantified HF counts for this and all time points looking at all HF PGRP-S-dsRed expression patterns to confirm this observation (Figure 4F); moreover, by quantifying only the SG-PGRP signal, we identified a significant induction peak at the 72h post exposure time point compared to all other time points (Figure 4G). Thus, expression of PGRP-S-dsRed in response to *S. aureus* appears to be specific to expression and secretion by the sebaceous gland of the hair follicle, suggesting the presence of activated epithelial cells at the HF-SG junction.

We further quantified expression levels of the dsRed across skin regions, by categorizing hair follicle PGRP-S-dsRed expression patterns expressed as percent PGRP+ and of those, SG-PGRP+. As a technical note, we did not directly quantify HS-PGRP so HFs that were PGRP-S+ but not SG-PGRP-S+ were depicted as the gray portion of the bar; therefore while the black portion of the bar in the figure includes any SG-PGRP + HF, it also contains dual expressing HFs. We found that the tail had the highest proportion of dsRed + HFs at 50% with a vast majority being SG-PGRP positive. Dorsal skin showed comparable levels of expression with almost 40% of hair follicles expressing some dsRed; however, only 18% showed the SG-PGRP pattern. Ear skin had the lowest percentage with dsRed expression in only 27% of follicles, the majority being SG-associated. (Figure 4H). Overall, this data reveals that skin at different anatomical locations showed region-specific patterns of dsRed expression in the HF.

### 3.4 Bulk gene expression data does not identify immune responses to acute, topical *Staphylococcus aureus* exposure

**FIGURE 5:**
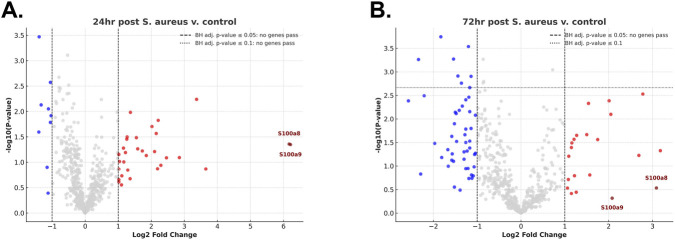
Bulk gene expression data does not identify immune responses to acute, topical Staphylococcus aureus exposure. **(A)** Volcano plots of gene expression data (-log (p-value) vs. log2 fold change) at 24 h and **(B)** 72 h post topical *S. aureus* exposure compared to baseline naïve control animals using the Nanostring nCounter Myeloid Innate Immunity Panel. Adjusted p-value thresholds (horizontal lines) set using Benjamini–Hochberg method for false discovery rate. Genes S100a8 and S100a9 are highlighted in red.

In the context of the microscopy data suggesting a rapid induction of antimicrobial responses to cutaneous *S. aureus* exposure, we next used a bulk gene expression approach to identify any additional immune genes regulated by the exposure. Interestingly, no genes were found to be significantly regulated after 24 or 72 h post *S. aureus* application when compared to naïve animals (Figure 5; Supplemental Information). Although we noted a high log fold change in S100a8 and S100a9, genes associated with neutrophil responses were not statistically significant using adjusted P values. We also screened for Pglyrp1 (PGRP-S) in this data set; however, it was pruned from analysis for low expression in all timepoints analyzed. It is likely that while some unidentified genes might yet be induced by microbial exposure, the use of a bulk gene expression profiling method caused any small or localized signal to be swamped out by the bulk of skin tissue cells that had little or no response. Indeed, this outcome highlights the power of our histological method to identify induction by less stimulating interventions and effects in a small minority population of cells.

## 4 Discussion

The PGRPs show distinct patterns of expression and regulation extending to the skin. The genes coding for PGRP-Iα and PGRP-Iβ are located on the epidermal differentiation complex (EDC) so constitutive expression in skin epithelia logically follows and has been demonstrated in both humans and mice (Mathur et al., 2004; Lu et al., 2006; Dziarski and Gupta, 2010; Park et al., 2011a). PGRP-L and PGRP-S, on the other hand, are seemingly inducible in the epidermis. PGRP-L has been found to be upregulated and protective in the context of bacterial exposure and psoriasis models (Wang et al., 2005; Park et al., 2011b). Importantly, this protein shows differential regulation of expression dependent on anatomical location, and presumably, constitutive versus induced states (Li et al., 2006). In this study, we reveal a novel expression pattern of PGRP-S in skin epithelia.

Our lab first established epithelial PGRP-S expression as a marker for microfold cells (M cells) in murine Peyer’s patches and advanced this finding into a tool to study these cells, the PGRP-S-dsRed reporter mice (Lo et al., 2003; Wang et al., 2011). Applied to other specialized epithelial beds such as nasopharyngeal-associated lymphoid tissue (NALT) and even thymus, this reporter identified these unique subsets of cells (Sakhon et al., 2015; Givony et al., 2023). In canonical M cells—those associated with the follicle associated epithelium (FAE) of established lymphoid follicles such as Peyer’s patches and NALT—PGRP-S-dsRed expression is constitutive.

The skin differs from mucosal tissues in many physical characteristics but similarly fields a continuous interplay between pathogens and host defense mechanisms. Here, HFs are the frontline. Viable bacteria have been shown to preferentially localize within follicles and invaginations with one study finding roughly one-quarter of all skin-associated bacteria being follicle-derived (Lange-Asschenfeldt et al., 2011; Acosta et al., 2023). Thus, the hair follicle forms a specialized microenvironment that mediates both microbial colonization and immune signaling making it a unique hub for immune coordination including this reported PGRP-S-dsRed expression. In this study, we uncover the presence of constitutive PGRP-S-dsRed + cells in the mouse vibrissae. Their localization to the inner epidermal layer of the isthmus places them at an ideal junction for antigen sampling functions reminiscent of mucosal M cells therefore, we sought to identify these cells as possibly M cell-like in nature since dsRed-expression using this reporter has been specific to this phenotype in all epithelial beds tested. First, PGRP-S+ cells stained positively for SpiB, the prototypical regulator of M cell differentiation (Kanaya et al., 2012). Second, they are close in proximity to possible APCs as identified by a CX3CR1-eGFP reporter. Although as mentioned, the whisker lacks a direct sub-epithelial lymphoid tissue, the presence of multiple layers of CX3CR1+ cells may be the bridge linking PGRP-S+ cells to a possible conduit for antigen and cell movement—the blood sinuses.

Interestingly, in the gut, an inducible M cell phenotype has also been described and termed “villous M cells” for their appearance on villi outside of the Peyer’s patches which were induced by stimuli such as dextran sodium sulfate (DSS) and *Citrobacter rodentium*. These cells were also notably identified through PGRP-S-dsRed expression (Bennett et al., 2016; Parnell et al., 2017). Similarly, others have shown PGRP-S to be inducible in response to trauma or inflammatory stimuli in other tissues. While absent in the lung epithelia of naïve mice, PGRP-S is induced in asthmatic, HDM (house dust mite)-sensitized mice (Park et al., 2013). We have also noted PGRP-S expression using our reporter in the airway lung epithelia using a transgenic model of global TNF overexpression (Figure 1A). Therefore, we can reliably confirm that specialized epithelial subsets—whether constitutive or induced—regulate PGRP-S expression distinctly from surrounding cells and we are able to utilize this expression pattern as an indication the initiation of an inflammatory or specialized epithelial program. Importantly, PGRP-S expression is distinctly regulated in mouse versus human tissues. While PGRP-S has been shown to be upregulated in response to synthetic PAMPs in human oral epithelial cells, human adenoid M cells do not show the characteristic expression seen in the analogous NALT of mice (Uehara et al., 2005; Sakhon et al., 2015; Alvarez-Arguedas et al., 2025). Accordingly, PGRP-S expression in mouse epidermal hair follicles might not be generalizable to human skin, even accounting for significant differences between different mouse epidermal regions (e.g., ear, the fur of flank skin, vibrissae, etc.) and human skin.

**FIGURE 6:**
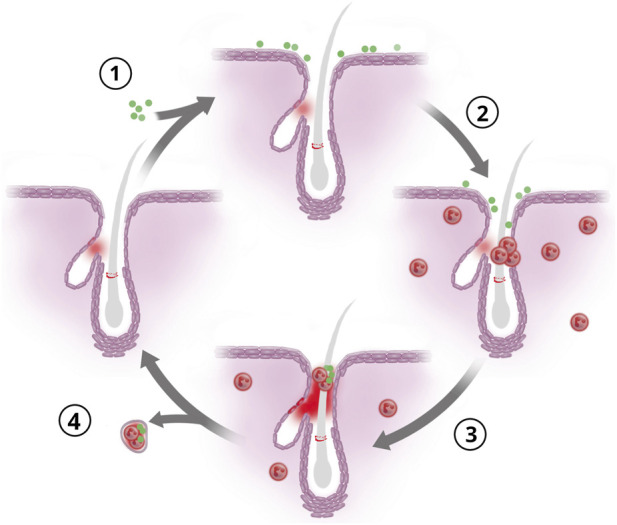
Model of host-pathogen interactions in the skin. 1. An “at rest” hair follicle comes into contact with a pathogenic microbe. 2. This results in either active or passive accumulation of microbes to the HF and dermal neutrophil recruitment which can create microabscess-like structures as a result of crossing into the HF lumen (hours to 1day). 3. Dermal neutrophil levels decreased to near baseline, however, PGRP-S secretion into the HF lumen has peaked. In some hair follicles, a “clot” of neutrophils, secretion and *Staphylococcus aureus* can be seen (around day 3). 4. The combination of neutrophilic microabscesses secretion milieu has mostly resolved and as early as 72 h, we can see these clot structures expelled.

In the dorsal skin, PGRP-S is not expressed at baseline to a significant degree, but we demonstrate a spatially and temporally restrained expression increase as a result of *S. aureus* topical application in mice. Importantly, it is anatomically localized to the hair follicle. This induction is specific to SG secretion in the HF (Figure 6) which differs from the expression in the whisker. While we confirmed the early neutrophil infiltration after 24 h of exposure, the SG-associated PGRP-S-dsRed followed but with a later peak around 72 h. The initial innate signaling provided by bacterial exposure may be common to neutrophil recruitment and hair follicle activation, but the difference in the kinetics of induction may reflect differences in the responding cells. The extremely rapid recruitment of neutrophils is through tissue expression of chemokines for a critical “first responder” protective response. In contrast, hair follicle expression of PGRP-S may represent a more coordinated synthesis of anti-microbial peptides along with other sebaceous gland products, providing secretions to cover and protect the opening of the anatomic niche. This PGRP-S containing “antimicrobial milieu” was evident in encapsulated microabscesses seen at the 72h timepoint. This stage represents a final phase for the innate protective mechanism as PGRP-S expression returns to baseline (Figure 6). It is also necessary to acknowledge PGRP-S as a multifunctional effector protein; yet the role of epithelial PGRP-S is largely unexplored. In corneal epithelium it was shown to be required for bacterial clearance and thought to act as a “broad spectrum deterrent.” (Ghosh et al., 2009) This may indeed be possible in the skin as we show much of the increased signal is secreted and in cells surrounding the sebaceous glands which are known active AMP producers (Zouboulis et al., 2022). However, the ability of pathogenic microbes to utilize M cells as points of entry indicates PGRP-S expressed in M cells may not have an AMP function, or at the very least M cell function does not rely on the AMP capabilities of PGRP-S in the way granulocytes do (Jensen et al., 1998; Dillon and Lo, 2019).

Although differences exist between the skin of humans and mice they are both highly colonized with Gram positive bacteria, and as the leading cause of skin and soft tissue infections (SSTIs), *S. aureus* is a clinically relevant microbe which has been widely used in models of cutaneous disease (McCaig et al., 2006; Lange-Asschenfeldt et al., 2011). However, many studies overlook effects in specialized structures such as HFs. Infection models often rely on intradermal or subcutaneous bacterial injection, which bypass the earliest host-microbe interactions at the epidermal keratinocyte barrier (Bitschar et al., 2017). Further, even when insults are applied topically, they can miss localized effects. One study looking at PGRPs in a model of dermatitis had shown increased levels of PGRP-S (PGLYRP1) in the skin as a result of topical oxazolone but attributed this to neutrophil recruitment to the skin (Park et al., 2011a). Similarly, our gene expression analysis was unable to detect transcriptional changes. Despite clear induction of PGRP-S at the protein level as seen through microscopy, bulk RNA from exposed skin showed no significant changes even though high log fold change from select inflammatory genes (S100a8 and S100a9) point to the neutrophil recruitment and are validated by the counts and images presented here. This result may be reflective of the rare, spatially restricted and transient nature of the events studied here. Exposure timing can also be an important factor to consider. Here, we look at acute microbial insult which highlights the rapid inducibility of the response and revealed the localization to the HF, however, topical exposure studies using chronic or repeated applications have been shown to lead to keratinocyte proliferation and broad transcriptomic responses (Wanke et al., 2013; Gribonika et al., 2025; Kline et al., 2025). Therefore, it would be logical next step to identify if these changes may also include more robust and longer lasting changes to the follicle epithelium such as resulting in a phenotype more similar to that of the vibrissae.

The native features and functions of the whisker also introduce a layer of complexity to this system. The hair follicle is poised at a unique interface, harboring a distinct development and turnover (the hair cycle), and more evidently serves as an important environment-sensing organ through the relaying of mechanotransduction signals. Based on our findings, however, the whisker follicle may be more versatile in its role. The presence of M cell-like cells points to previously undescribed immune sensing capabilities. A similar finding in the CVP taste buds identified a surveillance function in type 2 taste cells—cells specialized for sweet and umami flavors—therefore, studies were conducted to assess the interplay between these cells and the taste sensation. They found that mice lacking the M cell program through SpiB knockout showed altered preferences for these flavors (Qin et al., 2023). Therefore, the possible interplay between these roles could be reflected in vibrissae as well in some yet undetected manner and introduce a more intricate framework of “sensing” the surroundings.

### 4.1 Conclusion

Using PGRP-S-dsRed to identify specialized epithelial subtypes in the skin, we reveal distinct patterns of expression specific to anatomical region, but consistent in localization at hair follicles. While the whisker follicle seems poised to act as an immune-sensing organ, harboring M cell-like cells, dorsal skin follicles have a unique burst of expression upon microbial stimulation making activation of this phenotypic program a hallmark of hair follicle surveillance in cutaneous host defense. Together, our findings point to a stark phenotypic resemblance between epithelial beds suggesting a convergence in the demands at barrier tissues.

## Data Availability

The original contributions presented in the study are included in article/supplementary material. Further inquiries can be directed to the corresponding author/s.
